# Phosphorylation of *Mycobacterium tuberculosis* ParB Participates in Regulating the ParABS Chromosome Segregation System

**DOI:** 10.1371/journal.pone.0119907

**Published:** 2015-03-25

**Authors:** Grégory Baronian, Katarzyna Ginda, Laurence Berry, Martin Cohen-Gonsaud, Jolanta Zakrzewska-Czerwińska, Dagmara Jakimowicz, Virginie Molle

**Affiliations:** 1 Laboratoire de Dynamique des Interactions Membranaires Normales et Pathologiques, Universités de Montpellier II et I, Centre National de la Recherche Scientifique, UMR 5235, Montpellier, France; 2 University of Wroclaw, Faculty of Biotechnology, Department of Molecular Microbiology, Poland; 3 Centre de Biochimie Structurale, Centre National de la Recherche Scientifique, UMR 5048, Universités Montpellier I et II, Montpellier, France; University of Strathclyde, UNITED KINGDOM

## Abstract

Here, we present for the first time that *Mycobacterium tuberculosis* ParB is phosphorylated by several mycobacterial Ser/Thr protein kinases *in vitro*. ParB and ParA are the key components of bacterial chromosome segregation apparatus. ParB is a cytosolic conserved protein that binds specifically to centromere-like DNA *parS* sequences and interacts with ParA, a weak ATPase required for its proper localization. Mass spectrometry identified the presence of ten phosphate groups, thus indicating that ParB is phosphorylated on eight threonines, Thr32, Thr41, Thr53, Thr110, Thr195, and Thr254, Thr300, Thr303 as well as on two serines, Ser5 and Ser239. The phosphorylation sites were further substituted either by alanine to prevent phosphorylation or aspartate to mimic constitutive phosphorylation. Electrophoretic mobility shift assays revealed a drastic inhibition of DNA-binding by ParB phosphomimetic mutant compared to wild type. In addition, bacterial two-hybrid experiments showed a loss of ParA-ParB interaction with the phosphomimetic mutant, indicating that phosphorylation is regulating the recruitment of the partitioning complex. Moreover, fluorescence microscopy experiments performed in the surrogate *Mycobacterium smegmatis ΔparB* strain revealed that in contrast to wild type Mtb ParB, which formed subpolar foci similar to *M*. *smegmatis* ParB, phoshomimetic Mtb ParB was delocalized. Thus, our findings highlight a novel regulatory role of the different isoforms of ParB representing a molecular switch in localization and functioning of partitioning protein in *Mycobacterium tuberculosis*.

## Introduction

*Mycobacterium tuberculosis* (*M*. *tb*), the etiologic agent of Tuberculosis, infected 8.6 million people and was responsible for the death of 1.3 million people in 2012 thus remaining a major threat to global health [1]. The emergence of multi-drug-resistant strains makes *M*. *tb* an increasing global health threat since the number of newly infected people, emphasized by AIDS infections, keeps increasing in both developing and industrialized countries (WHO). *M*. *tb* has a complex lifestyle that relies on its remarkable capacity to survive within infected host macrophages by modulating its own cell functions in order to adapt to a new environment. One of the remarkable features of mycobacteria, crucial for the persistent infection, is their ability to enter dormant, non-replicating state. However, little is known about the control of mycobacterial cell cycle.

In bacteria, the key processes of the cell cycle, chromosome replication and segregation, are tightly coupled. Unlike in eukaryotes, bacterial chromosome segregation occurs during ongoing replication. The process of replication begins in a defined *locus*, called origin of replication (*oriC*). Newly duplicated o*riC* regions are precisely organized, moved and anchored by the segregation machinery. Subsequently, the rest of chromosome(s) is actively transported into specific positions in future daughter cells. In many bacteria, both organization and segregation of *oriC* regions are governed by the ParABS partitioning system. ParBs are DNA binding proteins with specific affinity for centromere-like *parS* sequences; ParA homologues are cytoskeletal weak ATPases directly interacting with ParB and proposed to be the motor element of the segregation machinery via polymerization/depolymerization events [2]; and centromere-like *parS* sequences are palindromic sequences of 14–16 mers located in the vicinity of the *oriC* region.

By interacting with *parS* and ParA, ParB promotes the formation of a core structure called the segrosome where other proteins participating in chromosome segregation can dock. This in turn promotes oligomerization to form a larger nucleoprotein complex whose movement is governed by a cytoskeletal ParA protein [3]. The *parB* homologues are present in most bacterial genomes (with exception of *E*. *coli*); in *Caulobacter crescentus* [4,5], *Streptomyces coelicolor* [6,7], *Corynebacterium glutamicum* [8], *Pseudomonas spp*. [9–11], *Myxococcus xanthus* [12,13] and *Bacillus subtilis* [14–17] are non-essential for almost all bacteria except *Caulobacter crescentus* [4] and *Myxococcus xanthus* [13] while in the other species elimination of ParB leads to severe chromosome segregation defects and associated with an increased number of anucleate cells [8,9,13,18]. The segrosome is thought to organize and position the *oriC* proximal part of the chromosome at the cell pole of the bacterium before cell division.

While replication [19–22] and cell division [23–26] are relatively well-studied processes in mycobacteria, chromosome segregation remains to be fully understood. Previously it was shown that mycobacterial chromosomes encode proteins participating in chromosome segregation [6,27,28]. Interestingly, transposon mutagenesis experiments revealed that *parB* could also be essential in *Mycobacterium tuberculosis* H37Rv [29], but it was proved to be nonessential in *M*. *smegmatis* [30]. *M*. *smegmatis parB* mutant strain produced increased number of anucleate cells. In addition, microscopy analysis revealed that proper positioning of ParB in the cytosol of *M*. *smegmatis* depends on the presence of ParA thus highlighting the fact that the functional ParABS system is crucial to accurate chromosome segregation in mycobacteria [31]. Moreover, *in vitro* experiments showed that *M*. *tuberculosis* ParB interacts with *parS* sequences and ParA, which would enhance ParB affinity for *parS* sequences.

Understanding *M*. *tuberculosis* (*M*. *tb*) cell cycle is crucial to decipher the mechanism leading to its remarkable persistence within host cells. This persistence is a unique feature enabling *M*. *tb* to modulate its own cell functions in order to adapt to new environmental conditions. A well-known mechanism of signal transduction in prokaryotes and eukaryotes is phosphorylation. The Ser/Thr Protein Kinases (STPKs) have been shown to regulate major cell processes so that Ser/Thr phosphorylation has emerged as a key regulatory process in mycobacteria [32,33]. Cell division is a key cell process that undergoes a tight regulation either in time and space. Interestingly, recent studies identified several mycobacterial cell cycle proteins as mycobacterial STPKs substrates including the polar determinant DivIVA homologue named Wag31 [34], FtsZ [35], or FipA [36], a FtsZ partner. In this study, we show for the first time that *M*. *tb* partitioning protein ParB activity is regulated by Ser/Thr phosphorylation and that phosphorylation not only negatively affects ParB affinity for its specific centromere-like *parS* sequences, but also inhibits ParB interaction with its cytoskeletal partner ParA. Furthermore, our microscopy observations suggest that phosphorylation also triggers ParB delocalization. Consequently, phosphorylation of ParB via Ser/Thr kinase phosphorylation would inhibit the ParABS system and thus the formation of the mitotic-like apparatus, which is essential to proper chromosome segregation.

## Materials and Methods

### Bacterial strains and growth conditions

Strains used for cloning and expression of recombinant proteins were *E*. *coli* 10G (Lucigen) and *E*. *coli* BL21(DE3)Star (Novagen) as detailed in S1 Table. They were grown in LB medium at 37°C. Media were supplemented with ampicillin (100 μg/ml), hygromycin (200 μg/ml), kanamycin (25 μg/ml) or spectinomycin (100 μg/ml) when required. Mycobacteria strains were grown aerobically on Middlebrook 7H10 agar plates with OADC enrichment (Difco) or in Middlebrook 7H9 medium supplemented with 10% (v/v) OADC enrichment, 0.5% (v/v) glycerol and 0.05% (v/v) Tyloxapol. Hygromycin (50 μg/ml) and kanamycin (25 μg/ml) were added for the selection of appropriate strains.

### Cloning, expression and purification of M. tuberculosis ParB and mutant proteins

The *parB* gene was amplified by PCR using *M*. *tb* H37Rv chromosomal DNA as the template and the forward and reverse primers listed in S2 Table containing *Nde*I and *Hind*III restriction sites, respectively. The amplified product was digested with *Nde*I and *Hind*III, and ligated into the pETPhos plasmid [37], a variant of pET15b (Novagen) that includes a tobacco etch virus (TEV) protease site instead of the thrombin site, and an N-terminal His-tag free of Ser/Thr/Tyr residues, thus generating pETPhos_*parB*. The *parB* gene containing either the ten mutations T32A, T41A, T53A, T110A, T195A, T254A, T300A, T303A, S5A, S239A, or the mutations T32D, T41D, T53D, T110D, T195D, T254D, T300D, T303D, S5D, S239D, were both synthesized by GenScript with 5' *Nde*I and 3' *Hind*III restriction sites. These two *parB* mutants were cloned into the pETPhos vector, generating pETPhos_*parB_* T32A/T41A/T53A/T110A/T195A/T254A/T300A/T303A/S5A/S239A and pETPhos_*parB_* T32D/T41D/T53D/T110D/T195D/T254D/T300D/T303D/S5D/S239D named pEThos_*parB_Ala* and pETPhos_*parB*_*Asp*, respectively. A duet strategy was used to generate hyper-phosphorylated ParB protein as described previously [38]. The *parB* gene was cloned into the pCDFDuet-1 vector already carrying the sequence encoding the PknB kinase domain using primers listed in S2 Table, thus generating plasmid pDuet_*parB*, which was used to transform *E*. *coli* BL21(DE3)Star cells in order to overexpress His-tagged ParB. All constructs were verified by DNA sequencing. Recombinant strains harboring the different constructs were used to inoculate 400 ml of LB medium supplemented with ampicillin or spectinomycin, and the resulting cultures were incubated at 37°C with shaking until the optical density of the culture reached an *A*_600_ of 0.6. IPTG (0.5 mM final) was added to induce the overexpression, and growth was continued for 3 h at 37°C. Purification of the His-tagged recombinant proteins was performed as described by the manufacturer (Clontech).

### In vitro kinase assays

*In vitro* phosphorylation was performed with 4 μg of wild-type ParB or ParB derivatives in 20 μl of buffer P (25 mM Tris-HCl, pH 7.0; 1 mM DTT; 5 mM MgCl_2_; 1 mM EDTA; 50μM ATP) with 200 μCi ml^-1^ (65 nM) [γ-^33^P]ATP (PerkinElmer, 3000 Ci mmol^-1^), and 2 to 4 μg of kinase in order to obtain the optimal autophosphorylation activity for each mycobacterial kinase for 30 min at 37°C. Each reaction mixture was stopped by addition of an equal volume of 5 × Laemmli buffer and the mixture was heated at 100°C for 5 min. After electrophoresis, gels were soaked in 16% TCA for 10 min at 90°C, and dried. Radioactive proteins were visualized by autoradiography using direct exposure to films.

### Mass spectrometry analysis

Purified His-tagged hyper-phosphorylated ParB (ParB-P) from the *E*. *coli* strain carrying pDuet_*parB* and co-expressing the PknB kinase domain, was subjected to mass spectrometry without further treatment. Subsequent mass spectrometric analyses were performed as previously reported [39–42]. Spectra were analyzed with the paragon algorithm from the ProteinPilot 2.0 database-searching software (Applied Biosystems) using the phosphorylation emphasis criterion against a homemade database that included the sequences of ParB and its derivatives.

### Electrophoretic mobility shift assay

The DNA probe for electrophoretic mobility shift assays (EMSA) was generated by PCR using *M*. *tb* H37Rv chromosomal DNA as a template which encompassed the *parS* region with respective primers pair listed in S2 Table. The 5' end of the double-stranded PCR product was labeled using [γ-^32^P]-ATP and T4 polynucleotide kinase. A typical assay mixture contained in 20 μl: 10 mM Tris-HCl, pH 7.5; 50 mM NaCl; 1 mM EDTA; 1 mM dithiothreitol (DTT); 0.1 μg of nonspecific competitor (polydI-dC); 5% (v/v) glycerol; radioactive DNA probe (2000 cpm.ml^-1^) and various amounts (0,5 to 2,5μM) of the purified ParB proteins. After 20 min of incubation at room temperature, 20 μl of this mixture was loaded onto a native 5% (w/v) polyacrylamide TBE Ready Gel (Bio-Rad) and electrophoresed in 1% TBE (v/v) buffer for 1 h at 100 V.cm^-1^. Radioactive species were detected by autoradiography using direct exposure to films.

### Overexpression of ParB and derivatives in M. smegmatis mc^2^155 and their purification

*M*. *tb parB* and *parB_Ala* genes were amplified from the corresponding pETPhos vector contructs and cloned into the shuttle vector pVV16 [43] using the primers listed in S2 Table. The resulting constructs pVV16_*parB* and pVV16_*parB_Ala* were electroporated into *M*. *smegmatis mc* ^*2*^ *155*. Transformants were grown and used for the purification of the His-tagged ParB proteins as described above. The purified recombinant proteins were used for immunoblotting using anti-phosphothreonine and anti-phosphoserine antibodies according to the manufacturer’s instructions (Invitrogen) and revealed using secondary antibodies labeled with IRDye 800CW infrared dyes (LiCOR).

### Bacterial two-hybrid assays

*M*. *tb parB*, *parB_Ala* and *parB_Asp* genes were amplified from the corresponding pETPhos vector contructs and cloned into the pUT18C or pKT25 bacterial two-hybrid vectors as previously described [44] using the primers listed in S2 Table. The resulting constructs were chemically transformed into *E*. *coli* BTH101 [44] cells. Transformants were plated onto LB/X-Gal media supplemented with ampicillin and kanamycin and incubated at 30°C for 48 h.

### Microscopy

*M*. *tb parB*, *parB_Ala* and *parB_Asp* genes were amplified from the corresponding pETPhos vector constructs and cloned into the pVV16_*egfp* vector with respective primers pair listed in S2 Table. The resulting constructs were electroporated into *M*. *smegmatis* mc^2^155Δ*parB* strain [30]. For protein localization, *M*. *smegmatis* mc^2^155Δ*parB* complemented strains were grown to OD600 0.8, fixed in 2% paraformaldehyde solution, washed with phosphate buffered saline (PBS; 10 mM sodium phosphate, pH 7.4, 150 mM NaCl, 15 mM KCl) and placed on poly-L-lysine coated coverslips before slides mounting using Vectashield H1000 (Vector). Fixed cells with fluorescent ParB were observed with an AxioImager Z2 (Zeiss) equipped with an apotome, using a 63X NA 1.4 apochromat DICIIIPhIII objective. Images were acquired and processed with the Zen software (Zeiss). For the detection of ParB-GFP derivatives, cytoplasmic protein extracts were isolated from *M*. *smegmatis* mc^2^155Δ*parB* complemented strains cell cultures grown until OD600 0.8 in 7H9 medium complemented with kanamycin (25μg/ml) at 37°C as previously described, and protein fractions (10 μg/lane) were separated using SDS-PAGE, blotted onto a nitrocellulose membrane, and subjected to Western blot analysis using the anti-GFP antibody (Santa Cruz).

## Results and Discussion

### ParB is phosphorylated in vitro by mycobacterial Ser/Thr protein kinases

The *M*. *tuberculosis* genome encodes eleven Ser/Thr protein kinases (STPKs) [45,46]. While these mycobacterial kinases appear to be involved in different key pathways such as cell wall metabolism, antibiotic susceptibility, and virulence [32,33,47,48], little is known about the nature of the target proteins that are phosphorylated.

**Fig 1:**
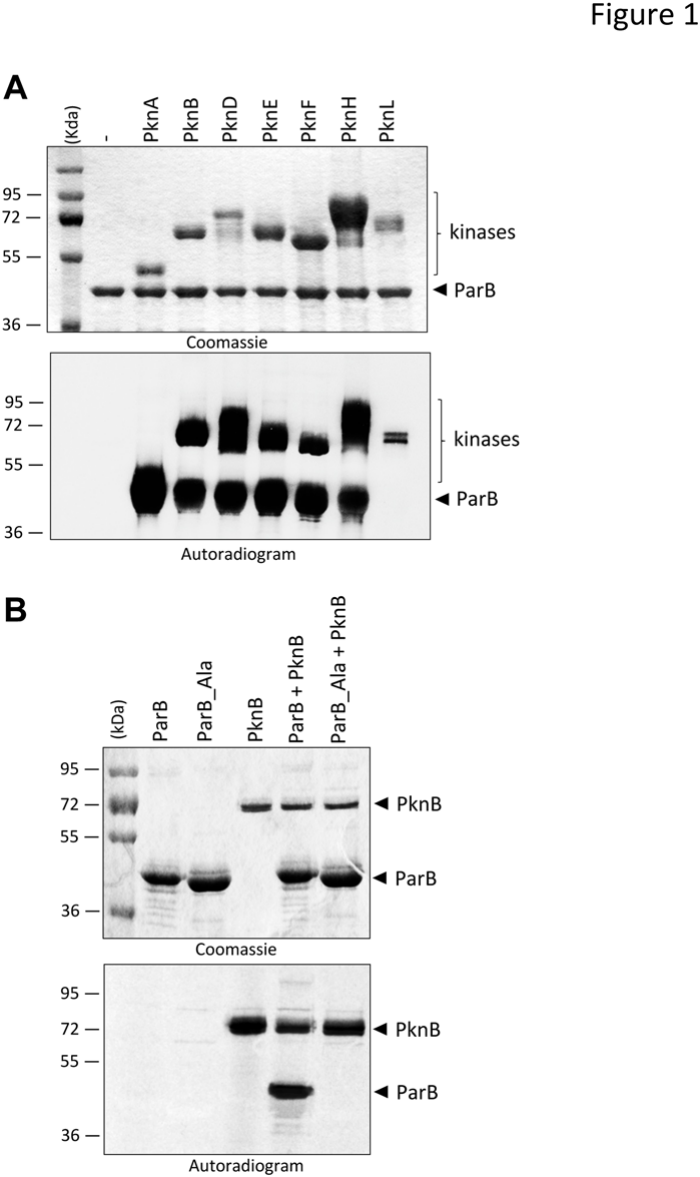
*In vitro* phosphorylation of ParB and mutant derivatives. **(A)** *in vitro* phosphorylation of *M*. *tb* ParB by PknB. The soluble domains of seven recombinant *M*. *tb* STPKs (PknA to PknL) were expressed and purified as GST-tagged fusions and incubated with purified His-tagged ParB and [γ-^33^P]ATP. The amount of the STPKs used varied from 0,3 to 2 μg to obtain the optimal autophosphorylation activity for each kinase. Samples were separated by SDS-PAGE, stained with Coomassie Blue (upper panel), and visualized by autoradiography after overnight exposure to a film (lower panel). Upper bands reflect the phosphorylation signal of ParB, and the lower bands correspond to the autophosphorylation activity of each kinase. *M*, molecular mass markers. **(B)** *in vitro* phosphorylation of the ParB_Ala mutant. Purified ParB and phosphoablative ParB (ParB_Ala) were incubated with PknB and [γ^33^-P]ATP. Samples were separated by SDS-PAGE, stained with Coomassie Blue (upper panel), and visualized by autoradiography (lower panel) after overnight exposure to a film.

We therefore decided to investigate whether the chromosome segregation system could be regulated by phosphorylation, and decided to test the major protein ParB. This was first investigated *in vitro* in the presence of purified STPKs. The soluble kinase domains of several transmembrane kinases from *M*. *tuberculosis* were expressed as GST-tagged fusion proteins and purified from *E*. *coli* as reported earlier [49]. The kinase enzymes were incubated with *M*. *tuberculosis* ParB and [γ-^33^P]ATP, the proteins were resolved by SDS-PAGE and the protein phosphorylation status was analyzed by autoradiography. The presence of an intense radioactive signal indicated that ParB was phosphorylated by PknA, PknB, PknD, PknE, PknF and PknH (Fig. 1A). No signal was observed in the presence of PknL. As expected, no radioactive band was observed in the absence of kinase. Another finding arising from these experiments is that ParB can interact with multiple STPKs as previously shown for *M*. *tuberculosis* STPK substrates [33], suggesting that these enzymes may be regulated by multiple signals. However, it remains to be established whether this STPK cross-talk occurs *in vivo*, which would argue for a very complex signalling mechanism.

### ParB is phosphorylated on serine and threonine residues

Mass spectrometry was used to identify and localize the phosphorylation site(s) on *M*. *tuberculosis* ParB. This method has been successfully used to elucidate the phosphorylation sites in a sequence-specific fashion for several *M*. *tuberculosis* STPK substrates [39,40,50–53]. Phosphorylated ParB-P was purified from *E*. *coli* co-expressing PknB and ParB (pDuet_*parB*) and subjected to mass spectrometric analysis after tryptic digestion. Noteworthy, we decided to use the PknB kinase previously described as involved in cell division processes among the kinases able to phosphorylate ParB in our *in vitro* assay [34,54,55]. A sequence coverage of 98% that included all Ser and Thr residues was obtained. The MS/MS spectra unambiguously identified the presence of ten phosphate groups (Table 1), thus indicating that ParB is phosphorylated on eight threonines, Thr32, Thr41, Thr53, Thr110, Thr195, and Thr254, Thr300, Thr303 and on two serines, Ser5 and Ser239. Moreover, in order to confirm that the phosphosites identified were not dependent on the kinase co-expressed with ParB, we performed mass spectrometry analysis when ParB was phosphorylated by PknF or PknH which resulted in the same phosphorylation sites identified. Most of the phosporylated residues are located in non-conserved regions of the ParB/Spo0J protein family, either in N- or C- terminus of the common conserved central domain (residues 60 to 251 in *M*. *tuberculosis* ParB) according to sequence alignment with the crystallised member ParB/Sp0J protein family, (*i*.*e*. Spo0J from *Thermus thermophiles* that shares 48% sequence identity with ParB for its core domain). The only phosphorylated residue positioned in the core domain is the threonine Thr195. This residue is located in the middle of one of the helix from the helix-turn-helix DNA-binding motif.

**Table 1:** Phosphoacceptors identified after purification of *M*. *tuberculosis* ParB from the *E*. *coli BL21(DE3)star* strain co-expressing *M*. *tuberculosis* PknB.

Phosphorylated tryptic peptide sequence of ParB purified from pCDFDuet co-expressing PknB	Number of detected phosphate groups (LC/MS/MS)	Phosphorylated residue(s)
[15–28] ENLYFQGHMTQP**pS**R	1	**S5**
[36–58] GLAALIPTGPADGESGPP**pT**LGPR	1	**T32**
[42–58] PTGPADGESGPP**pT**LGPR	1	**T32**
[59–83] MoxGSA**pT**ADVVIGGPVPDTSVM GAIYR	1	**T41**
[59–83] MGSA**pT**ADVVIGGPVPD**pT**SVM GAIYR	1	**T41+T53**
[59–83] MGSATADVVIGGPVPD**pT**SVMGAIYR	1	**T53**
[126–143] SLAGSQ**pT**GVRYQIVMGER	1	**T110**
[211–220] SRPLI**pT**NMIR	1	**T195**
[244–261] ALLSLEAGPEAQEELA**pS**R	1	**S239**
[244–270] ALLSLEAGPEAQEELA**pS**RIVAEGLSVR	1	**S239**
[271–285] ATEE**pT**VTLANHEANR	1	**T254**
[271–299] ATEE**pT**VTLANHEANRQAHHSDATTPAPPR	1	**T254**
[315–329] LSTTFD**pT**RVTVSLGK	1	**T300**
[315–330] LSTTFD**pT**RVTVSLGKR	1	**T300**
[315–330] LSTTFD**pT**RV**pT**VSLGKR	1	**T300+T303**
[315–329] LSTTFDTRV**pT**VSLGK	1	**T303**
[315–330] LSTTFDTRV**pT**VSLGK	1	**T303**

Sequences of the phosphorylated peptides identified in ParB as determined by mass spectrometry following tryptic digestion are indicated, and phosphorylated residues (pT or pS) are shown in bold.

Then, to confirm that only the identified residues are phosphorylated *in vitro*, these residues were mutated to alanine. The corresponding phosphoablative ParB_T32A/T41A/T53A/T110A/T195A/T254A/T300A/T303A/S5A/S239A mutant (ParB_Ala) was expressed as a His-tagged protein in *E*. *coli* BL21(DE3)Star harboring pETPhos_*parB_Ala*. The resulting ParB_Ala mutant protein was purified and incubated with PknB in the presence of [γ-^33^P]ATP. Following separation by SDS-PAGE and analysis by autoradiography, total abrogation of the phosphorylation signal was observed (Fig. 1B).

### ParB is phosphorylated in mycobacteria

**Fig 2:**
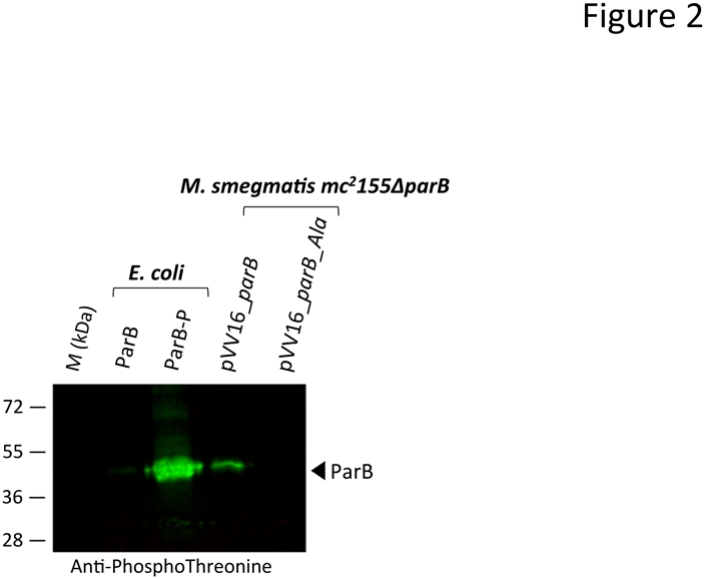
Phosphorylation of ParB in mycobacteria. *E*. *coli* harboring pETPhos_*parB* was used as a source of non phosphorylated ParB (ParB), and the strain harboring pDuet_*parB* coexpressing PknB and ParB provided the phosphorylated ParB isoform (ParB-P). ParB and ParB_Ala were produced in *M*. *smegmatis mc* ^*2*^ *155*Δ*parB* strains harboring pVV16_*parB* or pVV16_*parB*_*Ala*, respectively. Three μg of purified His-tagged ParB derivatives were migrated and detected on independent SDS-PAGE gels by immunoblotting using anti-phosphothreonine (middle panel) or anti-phosphoserine (lower panel) antibodies according to the manufacturer’s instructions (Invitrogen), and revealed with secondary antibodies labeled with IRDye infrared dyes (Odyssey, LiCOR). *M*, molecular mass markers.

To address the relevance of our *in vitro* findings, we investigated the phosphorylation status of *M*. *tb* ParB *in vivo*. To do so, we used the convenient *M*. *smegmatis mc* ^*2*^ *155* strain as a surrogate for *M*. *tb* as reviewed by Shiloh *et al*. [56]. First, due to the homology of ParB from *M*. *tb* and *M*. *smegmatis* (72%), we confirmed *in vitro* phosphorylation of *M*. *tb* ParB by PknB from *M*. *smegmatis* (data not shown). Next, we investigated the *in vivo* phosphorylation status by western blotting using either anti-phosphothreonine, or anti-phosphoserine antibodies [39,41]. First, the specificity of the antibodies was determined using the ParB isoforms purified from either *E*. *coli* or *E*. *coli* co-expressing PknB, based on the strategy described by Molle *et al*. (2010) [38]. Only the phosphorylated ParB isoform derived from pDuet_*parB* (ParB-P) reacted with the anti-phosphothreonine and antiphosphoserine antibodies as expected, while the unphosphorylated ParB isoform derived from the pETPhos_*parB* construct (ParB) failed to generate a signal with these antibodies (Fig. 2). Moreover, in order to address the role of ParB phosphorylation in mycobacteria, the genes encoding ParB and ParB_Ala (harboring Ser/Thr to Ala substitutions at ten positions as the phosphoablative control) were cloned as C-terminal His-tag fusions into the shuttle vector pVV16, and the resulting constructs, designated pVV16_*parB* and pVV16_*parB_Ala*, respectively, were introduced into *M*. *smegmatis mc* ^*2*^ *155*. The His-tag-purified proteins derived from these *M*. *smegmatis mc* ^*2*^ *155* recombinant strains were subjected to western blotting using anti-phosphothreonine, and anti-phosphoserine antibodies. As shown in Fig. 2, a clear signal for the wild-type ParB was detected with anti-phosphothreonine and anti-phosphoserine antibodies, while no signal was observed with the ParB_Ala (Fig. 2). No signal could be detected when probing the membrane with anti-phosphotyrosine antibodies (data not shown). Overall, these results confirm the presence of phosphorylated Ser and/or Thr residues and demonstrate that ParB from *M*. *tuberculosis* is indeed being phosphorylated in *M*. *smegmatis* thus confirming the relevance to use this *M*. *smegmatis* mutant in this study.

### Phosphorylation negatively regulates ParB DNA-binding to chromosome parS sequence

**Fig 3:**
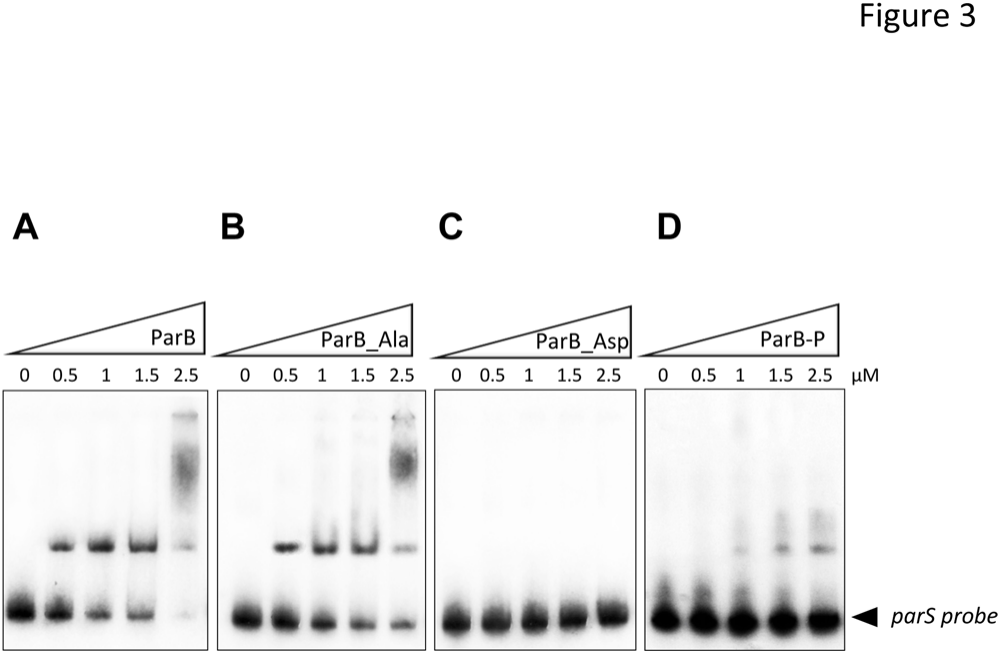
DNA-binding activity of ParB derivatives. Gel electrophoretic mobility shift analysis (EMSA) of ParB binding to the *parS* sequence. The *parS* region was amplified by PCR, radioactively labeled, and incubated with 0.5, 1, 1.5, and 2.5μM of purified ParB, resolved by non-denaturing PAGE and visualized by autoradiography after overnight exposure to a film. **(A)** Binding of the unphosphorylated ParB (ParB), **(B)** ParB phosphoablative mutant (ParB_Ala), **(C)** ParB phosphomimetic mutant (ParB_Asp), and **(D)** phosphorylated ParB (ParB-P), to the *parS* region.

Since ParB is described as a DNA-binding protein, we decided to investigate whether phosphorylation could affect ParB binding affinity to its chromosomal target sequence, the centromere-like *parS* sequences. To test this hypothesis, we analyzed and compared the ability of the non-phosphorylated (ParB) and the hyper-phosphorylated form (ParB-P) to bind to the *parS* region, known to be the specific target of ParB as previously described (Fig. 3) [30]. Electrophoretic mobility shift assays (EMSAs) were performed and revealed that the ParB unphosphorylated isoform (ParB) binds to the *parS* probe in a dose-dependent manner (Fig. 3A) as reported previously [30]. In contrast, the phosphorylated isoforms (ParB_Asp and ParB-P) showed a dramatic reduction of mobility shift (Fig. 3C and 3D), supporting the view that phosphorylation negatively affects the ability of ParB to bind to its chromosomal region. As anticipated, the ParB_Ala phosphoablative mutant retained a DNA-binding activity similarly to the non-phosphorylated protein (Fig. 3B). Our results clearly suggest that phosphorylation dramatically decreases ParB affinity for *parS* sequences at least *in vitro*. Therefore phosphorylation would negatively regulate ParB DNA-binding activity in mycobacteria, potentially by phosphorylation of the residue Thr195 located within the DNA binding domain. Therefore, a single T195D mutant was generated and while ParB_T195D binding was reduced it was not abolished, suggesting that the other phosphorylation sites are required for full regulation of ParB (data not shown).

### Phosphorylation inhibits ParB interaction with its cytoskeletal partner ParA

**Fig 4:**
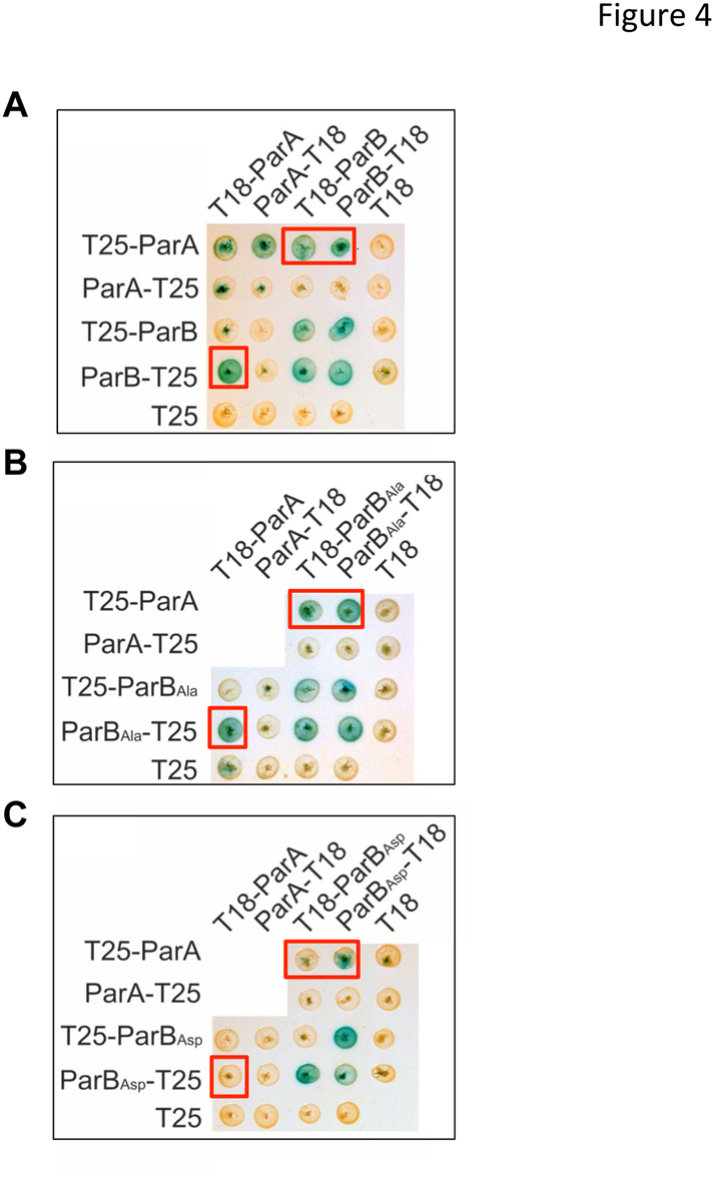
ParA interaction with ParB derivatives. Interactions of *M*. *tuberculosis* ParA with ParBWT **(A)** ParBAla **(B)** and ParBAsp **(C)** in bacterial two hybrid system. The red rectangles indicate differences in ParA-ParB interaction between WT (A), phosphoablative (B) and phosphophomimetic (C) ParB mutant proteins.

A recent study highlighted the essential role of ParA in the proper positioning and number of ParB complexes in *M*. *smegmatis* [31]. ParA localizes as pole-associated complexes connected with a patch of fluorescence accompanying two ParB complexes, positioned at 20–25% and 75–80% of the cell length. In the absence of ParA, ParB complexes are more numerous and seem to locate randomly into the cytosol highlighting the fact that ParB localization is tightly linked to its interaction with ParA. Based on these data and the localization of the phosphorylation sites along the primary sequence, we assessed the question, if phosphorylation could affect ParB interaction with ParA. This notion was verified by bacterial two hybrid (BTH) assays (Fig. 4). ParB derivatives and ParA were fused to either T25 or T18 fragment of *Bordetella pertussis* adenylate cyclase. Positive signals were observed with ParB_WT and ParB_Ala versus ParA, independently of the adenylate cyclase fragment and its position in the fusion protein suggesting that ParB_WT and ParB_Ala interact with ParA *in vivo*. Under the same conditions, no signal was observed when the interaction between ParB_Asp and ParA was tested indicating that ParB_Asp no longer interacts with ParA. Therefore, phosphorylation seems to also regulate ParB interaction with the cytoskeletal partner protein ParA. Noteworthy, BTH experiments indicated, that all ParB derivatives tested retain the ability to dimerise. This suggests, that replacement of the phosphorylation sites by alanine or aspartate does not prevent ParB dimerization, thus these results were not due to a tertiary or quaternary structure change due to the introduction of the mutations.

### Phosphorylation induces delocalization of ParB

**Fig 5:**
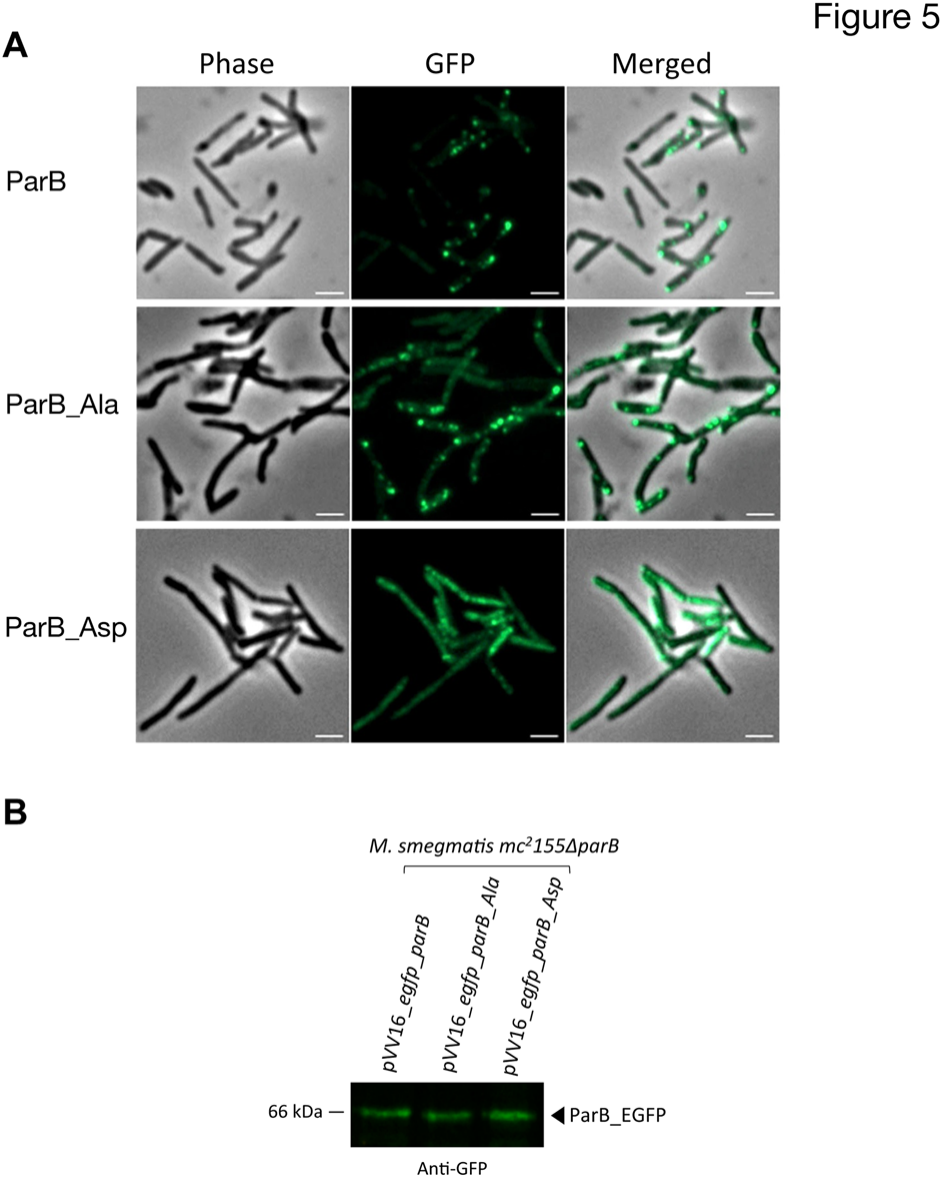
Localization of ParB and derivatives. **(A)** Subcellular localization of ParB-GFP in *M*. *smegmatis mc* ^*2*^ *155ΔparB* strain. Are shown Differential Intereference Contrast image (DIC), GFP fluorescence (GFP) and a merged image of DIC and GFP fluorescence (Merge). Localization of ParB isoforms are shown in three different panels: the upper panel shows wild-type ParB (ParB), the middle panel shows phosphoablative ParB (ParB_Ala) and the lower panel shows phosphomimetic ParB (ParB_Asp). Scalebar 2μm **(B)** Immunoblotting of ParB-GFP derivatives in *M*.*smegmatis mc* ^*2*^ *155ΔparB* complemented strains. Crude extracts of *M*. *smegmatis mc* ^*2*^ *155ΔparB* complemented with pVV16_*egfp_parB*, pVV16_*egfp_parB_Ala* or pVV16_*egfp_parB_Asp* were electrophoresed on SDS-PAGE gel, ParB-GFP derivatives were then detected by immoblotting using anti-GFP antibody according to the manufacturer’s instructions (Santa Cruz) and revealed with secondary antibodies labeled with IRDye infrared dyes (Odyssey, LiCOR).

Phosphorylation appears to prevent the interaction between ParB and ParA. Since ParB location is tightly dependent on the interaction with ParA we decided to investigate if phosphorylation could affect ParB complexes occurrence, number and/or localization *in vivo*. Wild-type ParB, phosphoablative and phosphomimetic ParB mutants were fused to EGFP using pVV16_*egfp* vector under *hsp60* promoter and the resulting constructs were introduced into *M*. *smegmatis mc* ^*2*^ *155*Δ*parB* strain [56]. *M*. *tuberculosis* ParB was fully functional in *M*. *smegmatis* cells, as demonstrated by the complementation of the *parB* deletion phenotype (2.52% anucleate cells, S3 Table). The localization of *M*. *tuberculosis* ParB resembled the native protein localization with two complexes in proximity of the cell poles (about 20 and 80% of cell length) (Fig. 5A). Such a positioning was observed in most analysed cells, however a fraction of cells with a diffuse fluorescence could also be observed. Both phosphomimetic and phosphoablative ParB mutant did not form clear foci in most of the cells but were mostly dispersed throughout the cell, either as a diffuse fluorescence (ParB_Asp) or irregularly spaced, faint foci (ParB_Ala) (Fig. 5A). This observation is in agreement with the lack of ParB_Ala and ParB_Asp complementation of segregation phenotype of *M*. *smegmatis parB* deletion mutant (S3 Table). While mislocalisation was expected in case of phosphomimetic mutant which does not bind DNA, it seems surprising for ParB_Ala. This suggests that although ParB_Ala is able to interact with DNA and ParA, the lack of phosphoregulation severely affects its localization and function. That could result from influence of phosphorylation on the dynamics of ParA-ParB-parS interactions. For example altered phosphorylation state of ParB could affect either stability of the nucleoprotein complex or ParB stimulated ParA ATPase activity changing its dynamics and localisation. Moreover, western blot analysis from ParB-GFP derivatives in *M*.*smegmatis mc* ^*2*^ *155ΔparB* complemented strains revealed proper expression of the GFP-recombinant ParB proteins in each strain (Fig. 5B).

We propose that both phosphorylated and unphosphorylated states of ParB are required for proper chromosome segregation and that a subtle balance between the phosphorylated and unphosphorylated states of ParB would tune the segregation event during the cell cycle.

## Conclusion

We have used a combination of genetic and biochemical approaches to provide the first reported evidence that the partitioning protein ParB, is negatively regulated by phosphorylation. Our work suggests that a shift in the unphosphorylated/phosphorylated ParB balance in favor of the phosphorylated form rapidly leads to the delocalization of ParB into the cytosol. An hypothesis arising from this work is that a regulatory role of the different isoforms of ParB represents a molecular switch in partitioning protein localization and function. The unphosphorylated form of ParB binds to its target DNA sequences, *parS*, and this binding is modulated via the interaction with the cytoskeletal partner protein ParA, which is thought to enhance the binding of ParB to its DNA targets if ParB is complexed with ParA. In addition, ParB interaction with ParA is critical for a proper positioning of ParB in proximity to the cell pole. The PknB-mediated phosphorylation of ParB inactivates the DNA binding activity of this partitioning protein, as well as the interaction with ParA, thereby preventing the localization of ParB, thus the formation of the mitotic-like apparatus. Our results provide a foundation for further investigation of an important functional linkage between STPKs and the ParABS chromosome segregation system, which may contribute to proper mycobacterial cell division. Interestingly, it was shown before that ParA interacts with another phosphorylated protein Wag31 (DivIVA homologue) possibly providing another link between segregation apparatus and STPKs regulation. Although challenging, future studies will help identify extracellular cues sensed by kinases leading to the phosphorylation of ParB. This will allow us to understand how *M*. *tuberculosis* senses its environment and mediates its response in a coordinated manner to regulate chromosome partition.

## Supporting Information

S1 Table Bacterial Strains and Plasmids used in this study. (DOCX)

S2 Table Primers used in this study. (DOCX)

S3 Table Frequencies of anucleate cells in *M*.*smegmatis mc* ^*2*^ *155ΔparB* complemented with fluorescent ParB derivatives compared to the wild type strain. (DOCX)
