# Supplementary material for: Phosphorylation of Mycobacterium tuberculosis ParB Participates in Regulating the ParABS Chromosome Segregation System
Source: PLoS One. 2015 Mar 25;10(3):e0119907. doi: 10.1371/journal.pone.0119907 (PMC4373775; doi:10.1371/journal.pone.0119907)
Supplement: S1 Table — (DOCX) [file pone.0119907.s001.docx]

**Supporting Information Table S1.** Bacterial Strains and Plasmids used in this study

| **Strains or Plasmids** | **Genotype or Description** | **Source or Reference** |  |
| --- | --- | --- | --- |
| ***E. coli* strains** |  |  |  |
| *E. coli* cloni10G | F- mcrA D(*mrr-hsd*RMS-*mcr*BC) f80*dlac*ZÄM15 D *lac*X74 *end*A1 *rec*A1*ara*D139 D (*ara*, *leu*)7697 *gal*U *gal*K *rps*L *nup*G - *ton*A ; *E. coli* derivative ultra competent cells used for general cloning. | Lucigen |  |
| *E. coli* BL21(DE3)Star | F^–^ *omp*T *hsd*SB(r_B_^–^, m_B_^–^)*gal dcm rne*131(DE3); used to express recombinant proteins | Stratagene |  |
| *E. coli* BTH101 | F–, *cya-99*, *araD139, galE15, galK16, rpsL1 (Str r)*, *hsdR2, mcrA1, mcrB1* used to perform BTH assay. | [1] |  |
| **Mycobacterial strains** |  |  |  |
| *M. smegmatis* mc^2^155 | *ept-1* | [2] |  |
| *M. smegmatis* mc^2^155*ΔparB* | *mc2155* strain deleted from *parB* | [3] |  |
| ***E. coli* plasmids** |  |  |  |
| pUC57 | pUC19 derivative, designed for cloning and generation of ExoIII deletions | Genscript |  |
| pUC57_*parB_Ala_* | pUC57 derivative used by Genscript to deliver synthesized *parB10Ala* gene where phosphorylated residues were substituted by Alanine (S5A/T32A/T41A/T53A/T110A/T195A/S239A/ T254A/T300A/ T303A) | This study |  |
| pUC57_ *parB_Asp_* | pUC57 derivative used by Genscript to deliver synthesized *parB10Asp* gene where phosphorylated residues were substituted by Aspartate (S5D/T32D/T41D/T53D/T110D/T195D/S239D/ T254D /T300D/ T303D) | This study |  |
| pETPhos | pET-15b (Novagen) derivative including the replacement of the thrombin site coding sequence with a tobacco etch virus (TEV) protease site and Ser to Gly mutagenesis in the N-term His-tag | [4] |  |
| pETPhos_*parB* | pETPhos derivative used to express His-tagged fusion of *Mycobacterium tuberculosis* ParB WT in *E. coli* | This study |  |
| pETPhos_*parB_Ala_* | pETPhos derivative used to express His-tagged fusion of  *Mycobacterium tuberculosis* ParB10Ala in *E. coli* | This study |  |
| pETPhos_*parB_Asp_* | pETPhos derivative used to express His-tagged fusion of  *Mycobacterium tuberculosis* ParB10Asp in *E. coli* | This study |  |
| pCDFDuet-1 | pET vector derivative designed for the co-expression of two proteins under *T7lac* promoter induction | Novagen |  |
| pDuet_*parB/pknB* | pET vector derivative used for the co-expression of PknB and His ParB proteins under *T7lac* promoter induction | This study |  |
| pDuet_*parB/pknF* | pET vector derivative used for the co-expression of PknF and His ParB proteins under *T7lac* promoter induction | This study |  |
| pDuet_*parB/pknH* | pET vector derivative used for the co-expression of PknH and His ParB proteins under *T7lac* promoter induction | This study |  |
| pKT25 | pSU40 derivative, allows expression of N-terminal T25 fragment (amino acids 1 to 224 of CyaA) fusion protein under *lac* promoter in *E. coli*. | [1] |  |
| pKNT25 | pSU40 derivative, allows expression of C-terminal T25 fragment (amino acids 1 to 224 of CyaA) fusion protein under *lac* promoter in *E. coli.* | [1] |  |
| pUT18 | pUC19 derivative, allows expression of N-terminal T18 fragment (amino acids 225 to 399 of CyaA) fusion protein under *lac* promoter in *E. coli*. | [1] |  |
| pUT18c | pUC19 derivative, allows expression of C-terminal T18 fragment (amino acids 225 to 399 of CyaA) fusion protein under *lac* promoter in *E. coli*. | [1] |  |
| pKT25_*parB_WT_* | pKT25 derivative used to express N-terminal CyaA T25 fusion of *M. tuberculosis* ParB WT in *E. coli* | This study |  |
| pKT25_*parB_Ala_* | pKT25 derivative used to express N-terminal CyaA T25 fusion of *M. tuberculosis* ParB Ala in *E. coli* | This study |  |
| pKT25_*parB_Asp_* | pKT25 derivative used to express N-terminal CyaA T25 fusion of *M.tuberculosis* ParB Asp in *E. coli* | This study |  |
| pKT25_*parA* | pKT25 derivative used to express N-terminal CyaA T25 fusion of *M. tuberculosis* ParA in *E. coli* | This study |  |
| pNKT25_*parB_WT_* | pNKT25 derivative used to express C-terminal CyaA T25 fusion of *M. tuberculosis* ParB WT in *E. coli* | This study |  |
| pNKT25_*parB_Ala_* | pNKT25 derivative used to express C-terminal CyaA T25 fusion of *M. tuberculosis* ParB Ala in *E. coli* | This study |  |
| pNKT25_*parB_Asp_* | pNKT25 derivative used to express C-terminal CyaA T25 fusion of *Mtuberculosis* ParB Asp in *E. coli* | This study |  |
| pNKT25_*parA* | pNKT25 derivative used to express C-terminal T25 of CyaA fusion of *M tuberculosis* ParA in *E. coli* | This study |  |
| pUT18_*parB_WT_* | pUT18 derivative used to express N-terminal CyaA T18 fusion of *M tuberculosis* ParB WT fused to in *E. coli* | This study |  |
| pUT18_*parB_Ala_* | pUT18 derivative used to express N-terminal CyaA T18 fusion of *M tuberculosis* ParB Ala in *E. coli* | This study |  |
| pUT18_*parB_Asp_* | pUT18 derivative used to express N-terminal CyaA T18 fusion of *M tuberculosis* ParB Asp in *E. coli* | This study |  |
| pUT18_*parA* | pUT18 derivative used to express N-terminal CyaA T18 fusion of *M tuberculosis* ParA in *E. coli* | This study |  |
| pUT18c_*parB_WT_* | pUT18c derivative used to express C-terminal CyaA T18 fusion of *M tuberculosis* ParB WT fused to in *E. coli* | This study |  |
| pUT18c_*parB_Ala_* | pUT18c derivative used to express C-terminal CyaA T18 fusion of *M tuberculosis* ParB Ala in *E. coli* | This study |  |
| pUT18c_*parB_Asp_* | pUT18c derivative used to express C-terminal CyaA T18 fusion of *M tuberculosis* ParB Asp in *E. coli* | This study |  |
| pUT18c_*parA* | pUT18c derivative used to express C-terminal CyaA T18 fusion of *M tuberculosis* ParA in *E. coli* | This study |  |
| **Mycobacterial shuttle plasmids** |  |  |  |
| pVV16 | *E. coli*/mycobacterial shuttle vector, allows expression of C-terminal His-tagged proteins, derived from pMV261 and containing the *hsp60* constitutive promoter | [5] |  |
| pVV16_*parB-egfp* | pVV16 derivative used to express C-terminal EGFP-tagged fusion of ParB in mycobacteria | This Study |  |
| pVV16_*parB_Ala_-egfp* | pVV16 derivative used to express EGFP-tagged fusion of ParB phosphorylation sites mutated in alanine residues (S5A/T32A/T41A/T53A/T110A/T195A/S239A/T254A/T300A/ T303A) in mycobacteria | This Study |  |
| pVV16_*parB_Asp_-egfp* | pVV16 derivative used to express EGFP-tagged fusion of ParB phosphorylation sites mutated in aspartate residues (S5D/T32D/T41D/T53D/T110D/T195D/S239D/T254D/T300D/ T303D) in mycobacteria | This Study |  |
