# Supplementary material for: Phosphorylation of Mycobacterium tuberculosis ParB Participates in Regulating the ParABS Chromosome Segregation System
Source: PLoS One. 2015 Mar 25;10(3):e0119907. doi: 10.1371/journal.pone.0119907 (PMC4373775; doi:10.1371/journal.pone.0119907)
Supplement: S2 Table — (DOCX) [file pone.0119907.s002.docx]

**Supporting information Table S2.** Primers used in this study ^a, b^

| **Primer Name** | **Genes** | **5' to 3' Sequence** |
| --- | --- | --- |
| pETPhos Nterm ParB | *parB* | TAATAGCTCATATGACCCAGCCGTCACGCAGAAAG (*Nde*I) |
| pETPhos Cterm ParB | *parB* | TATAAGCTTTTACAGAGCGTCCCTGTGCAGGCC (*HindIII*) |
| pETPhos Nterm ParB_S5A | *parB_10Ala* | TAATAGCTCATATGACCCAGCCG**GCA**CGCAGAAAG (*NdeI*) |
| pETPhos Nterm ParB_S5D | *parB_10Asp* | TAATAGCTCATATGACCCAGCCG**GAC**CGCAGAAAG (*Nde*I)  (*Nde*I) |
| pCFDuet-1 Nterm ParB | *parB* | TAATAGCTCCATGGACCCAGCCGTCACGCAGAAAG (*NcoI*) |
| pCFDuet-1 Cterm ParB | *parB* | TATAAGCTTTTACAGAGCGTCCCTGTGCAGGCC (*HindIII*) |
| pVV16 Nterm ParB-EGFP | *parB* | TAATAGCTCATATGACCCAGCCGTCACGCAGAAAG (*NdeI*) |
| pVV16 Cterm ParB-EGFP | *parB* | TATAGATCTcagagcgtccctgtgcaggcctttatc (*BglII*)  (*HindIII*) |
| pVV16 Nterm ParB_S5A-EGFP | *parB_10Ala* | TAATAGCTCATATGACCCAGCCG**GCA**CGCAGAAAG (*NdeI*) |
| pVV16 Nterm ParB_S5D-EGFP | *parB_10Asp* | TAATAGCTCATATGACCCAGCCG**GAC**CGCAGAAAG (*Nde*I) |
| Nterm *parS* probe | *parS* | gcgagtggagggacggcgtga |
| Cterm *parS* probe | *parS* | cgcatcgcacgttctgctgcagcgccg |
| ETH_MB_F |  | ACTCTAGAGATGACCCAGCCGTCACGCAGAAAG |
| ETH_MB_R |  | gcggtaccgccagagcgtccctgtgcaggc |
|  |  |  |

^a^ Restriction sites are underlined and specified in brackets.

**^b^** Bold bases indicate the change of an amino acid to alanine, or aspartate.

**Table S3.** Frequencies of anucleate cells in *M.smegmatis* *mc^2^155∆parB* complemented with fluorescent ParB derivatives compared to the wild type strain.

| mc^2^155 | WT | *∆parB* | *∆parB/*  ParB_WT-EGFP | *∆parB/*  ParB_Ala-EGFP | *∆parB/*  ParB_Asp-EGFP |
| --- | --- | --- | --- | --- | --- |
| Percentage of anucleate cells | 0.20% | 11.33% | 2.52% | 11.35% | 10.51% |

**REFERENCES**

1. Karimova G, Ullmann A, Ladant D (2000) A bacterial two-hybrid system that exploits a cAMP signaling cascade in *Escherichia coli*. Methods Enzymol 328: 59-73.

2. Snapper SB, Melton RE, Mustafa S, Kieser T, Jacobs WR, Jr. (1990) Isolation and characterization of efficient plasmid transformation mutants of *Mycobacterium smegmatis*. Mol Microbiol 4: 1911-1919.

3. Jakimowicz D, Brzostek A, Rumijowska-Galewicz A, Zydek P, Dolzblasz A, et al. (2007) Characterization of the mycobacterial chromosome segregation protein ParB and identification of its target in *Mycobacterium smegmatis*. Microbiology 153: 4050-4060.

4. Canova MJ, Kremer L, Molle V (2008) pETPhos: a customized expression vector designed for further characterization of Ser/Thr/Tyr protein kinases and their substrates. Plasmid 60: 149-153.

5. Jackson M, Crick DC, Brennan PJ (2000) Phosphatidylinositol is an essential phospholipid of mycobacteria. J Biol Chem 275: 30092-30099.
