# Supplementary material for: Phosphorylation of Mycobacterium tuberculosis ParB Participates in Regulating the ParABS Chromosome Segregation System
Source: PLoS One. 2015 Mar 25;10(3):e0119907. doi: 10.1371/journal.pone.0119907 (PMC4373775; doi:10.1371/journal.pone.0119907)
Supplement: S3 Table — (DOCX) [file pone.0119907.s003.docx]

**Supporting information Table S3.** Frequencies of anucleate cells in *M.smegmatis* *mc^2^155∆parB* complemented with fluorescent ParB derivatives compared to the wild type strain.

| mc^2^155 | WT | *∆parB* | *∆parB/*  ParB_WT-EGFP | *∆parB/*  ParB_Ala-EGFP | *∆parB/*  ParB_Asp-EGFP |
| --- | --- | --- | --- | --- | --- |
| Percentage of anucleate cells | 0.20% | 11.33% | 2.52% | 11.35% | 10.51% |
